# Preference and possible consumption of provided enrichment and bedding materials and disinfectant powder by growing pigs

**DOI:** 10.1186/s40813-021-00243-w

**Published:** 2022-01-04

**Authors:** Felicitas Koch, Janine Kowalczyk, Hans Mielke, Hans Schenkel, Martin Bachmann, Annette Zeyner, Peter Leinweber, Robert Pieper

**Affiliations:** 1 Department Safety in the Food Chain, German Federal Institute for Risk Assessment, Berlin, Germany; 2 Department Exposure, German Federal Institute for Risk Assessment, Berlin, Germany; 3 Institute of Animal Science, University of Hohenheim, Stuttgart, Germany; 4 Institute of Agricultural and Nutritional Sciences, Martin Luther University Halle-Wittenberg, Halle (Saale), Germany; 5 Faculty of Agriculture and Environmental Sciences, University of Rostock, Rostock, Germany

**Keywords:** Bedding material, Enrichment material, Disinfectant powder, Peat, Biochar, Straw, Pig, Preference testing, Mass spectrometry, n-alkanes

## Abstract

**Background:**

Domestic pigs have an evolutionary conserved exploratory behaviour. To comply with this requirement, the European Union aims at setting standards for appropriate enrichment materials for pigs (Council Directive 2008/120/EC). As recommended characteristics include ‘chewable’ and ‘edible’, pigs might also consume these materials (Commission Recommendation (EU) 2016/336), which are often additionally advertised to enhance lying comfort and hygienic conditions in stables. To date, a wide range of bedding, enrichment and disinfectant materials is available on the market to ensure environmental enrichment, a dry, hygienic environment or lying comfort. Previous studies revealed considerable amounts of undesirable substances in some of these materials possibly being a risk for food safety considering oral uptake by the animal. To determine interest and indicators for consumption of different types of materials by pigs during exploratory behaviour, a camera-assisted observational study with 12 female pigs (German Landrace) was conducted. We tested their preference for a disinfectant powder, peat, biochar and straw as reference material in a 4 × 6 factorial arrangement.

**Results:**

Pigs manipulated and consumed all offered materials. However, longest manipulation time per pig was observed for biochar (63 min/day) and peat (50 min/day) (p < 0.05). Analyses of the bulk molecular-chemical composition and n-alkanes and acid insoluble ash as markers in the materials and in faeces clearly revealed the consumption of these materials by pigs.

**Conclusions:**

Whether the consumption of considerable amounts together with certain levels of undesirable substances represents a risk for pig and consumer health could yet not be established. Future studies will address the quantitative contribution of undesirable substances by oral ingestion of bedding and enrichment materials and disinfectant powders to the daily feed ration.

## Background

In free-range husbandry, pigs spend most of their active time (70–80%) foraging, whereas the actual time for feed intake is rather low. Although differences in morphology and physiology of wild boar and domestic pigs are obvious, the foraging behaviour was highly preserved during domestication [1, 2]. For animal welfare it is crucial that the inherent need to explore their surroundings by rooting, sniffing, biting or chewing, is also met in pig husbandry systems with an often barren environment. Enrichment and bedding materials enable pigs to perform these natural behaviours, preventing redirected harmful exploratory behaviour (tail and ear biting) toward pen mates [3, 4]. In the European Union (EU), Directive 2008/120/EC aims at standardizing the provision of appropriate materials [5], which might not be the case beyond EU regulations [6]. A wide range of materials is available on the market to ensure environmental enrichment, a dry and hygienic environment or lying comfort. Most materials can fulfil more than one of these purposes.

Materials should be chewable and even edible, ideally containing beneficial nutrients [7]. Consequently, a possible consumption of provided bedding and enrichment materials as well as disinfectant powders by farm animals cannot be excluded and might be even desired. Multiple studies confirm the positive effect of bedding and enrichment materials on health and performance of pigs [8–10]. However, recent data suggest that these materials may contain considerable amounts of toxic metals, which are considered as undesirable substances in feed [11, 12]. Thus, ingestion of contaminated materials by pigs might pose a risk to animal and consumer health. To accomplish both, animal welfare and the safety of products of animal origin, the Codex Alimentarius, providing international standards for safety of feed and food, suggests treating these materials likewise to feed [13]. A possible inclusion of environmental enrichment, bedding and disinfectant materials as feed materials has been recently discussed [12].

According to the structure, moisture content and odour, pigs have different preferences for different types of materials [14–16]. Depending on the material type, van Barneveld [17] showed a consumption of bedding material by pigs up to 14% of the daily ration, for which they used alkane markers to identify and quantify material consumption relative to feed intake. Such markers are ideally non-toxic, indigestible, non-absorbable, stable and inert when passing the gastrointestinal tract. Their passage rate should be identical to that of the solid phase of ingesta. The markers should accurately be detectable in faeces and should have a predictable recovery rate preferably close to 100% [18, 19]. As internal markers, substances can be used that are naturally occurring in feed, enrichment or bedding materials (e.g. n-alkanes or acid insoluble ash), whereas external markers (e.g. titanium dioxide) must be supplemented to the diet [20]. Usually, such markers are used to estimate feed intake, diet composition (i.e. selection of feed components from the diet), nutrient digestibility or passage rate [21–23]. In the current study, we used three different internal markers to identify the possible consumption of provided enrichment and disinfectant materials by pigs. We selected peat as highly preferred enrichment material by pigs [14, 15] and disinfectant powder as material with the highest risk potential of containing high amounts of undesirable substances according to Koch et al. [12]. Based on the abovementioned requirements, we chose the mass spectral pattern obtained from non-targeted analyses of bulk organic matter using a soft-ionization mass spectrometry as well as the concentrations of long-chain n-alkanes and acid insoluble ash (AIA) as indicators of peat and disinfectant powder ingestion, respectively. Bedding materials like straw, peat and others are characterized by typical mass spectral patterns which are obtained from in-source-pyrolysis coupled with (soft) field ionization mass spectrometry [24]. By comparing the mass spectral patterns of peat and pigs’ faeces, it may be possible to estimate if peat has been taken up and to which extent peat-derived molecules left the digestive tract and are excreted. In analogy, n-alkanes can be a possible marker for peat intake. Aliphatic n-alkanes are a component of epicuticular and intracuticular waxes in plants [25]. Although they are prone to degradation, they can also be found in peat [26]. In disinfectant powders, the main compound are silicates. The high content of crude ash also reveals a higher content of AIA, being a reliable marker with a recovery rate close to 100% in some studies and thus a possible effective marker in detecting oral uptake of disinfectant powder by pigs [27, 28].

So far, few studies investigating the consumption of bedding and environmental enrichment material by pigs exist. However, undesirable substances in these materials might have an influence on animal health and safety of products of animal origin. The objective of our study was to investigate, which type of enrichment or disinfectant material pigs are most interested in (exploring duration and frequency), assuming this as the material to be most likely ingested by pigs. Additionally, appropriate markers to identify a consumption of provided materials by pigs shall be identified.

## Results

Pigs were in good health status throughout the observational study. Presentation of different test materials revealed no adverse effects. An average daily gain of 0.92 kg ± 0.22 kg and average daily feed intake of 1.85 ± 0.68 kg during the experimental period (including adaptation) resulted in a feed conversion ratio of 2.12 ± 0.66.

### Behavioural observations

**Fig. 1:**
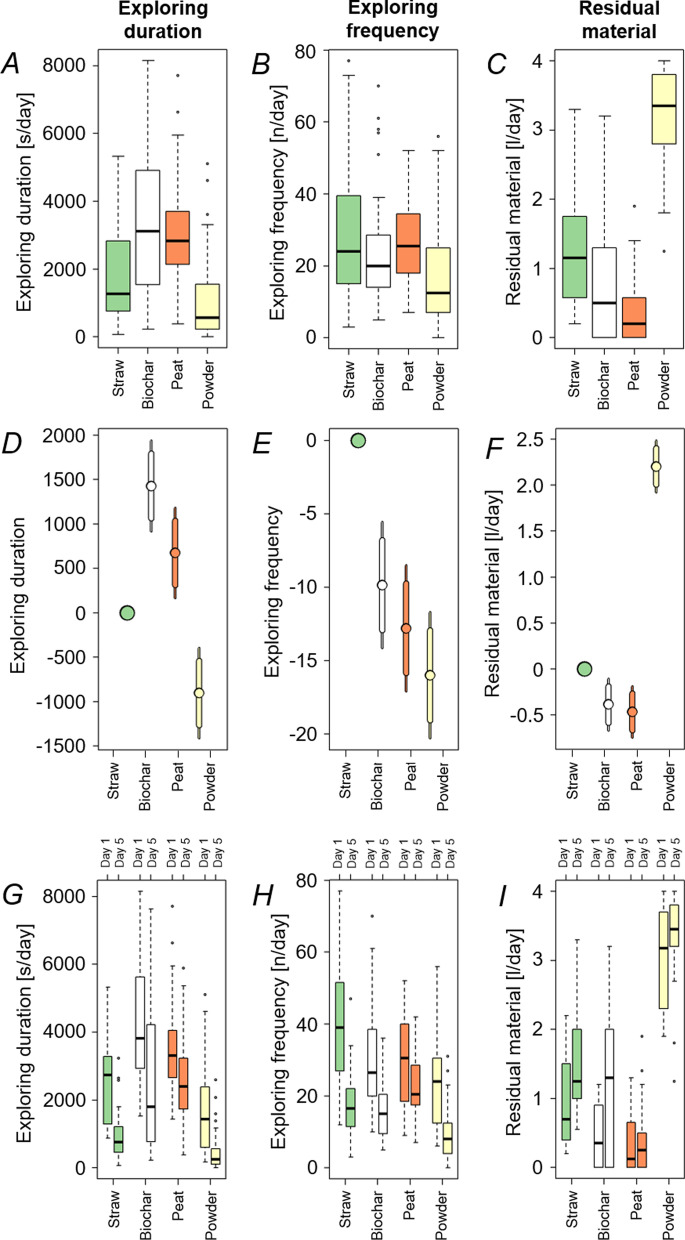
**A** Total duration and **B** frequency per pig (n = 12) exploring the materials and **C** residual material in the trough (n = 6); **D–F** 95% (narrow bar) and 84% (thick bar) confidence intervals, respectively—parameters differ significantly (p < 0.05) if 84% confidence intervals do not intersect; **G** duration and **H** frequency per pig (n = 12) exploring the materials and **I** residual material (*F*) in the trough (n = 6) shown for day one and five

Addressing the question which material pigs prefer most, statistical analysis focused on total duration and frequency of pigs exploring the material as well as residual material in the trough. Duration correlated clearly with frequency (r = 0.62, p < 0.05) as well as with residual material (r = − 0.61, p < 0.05). Correlation of exploring frequency and residual material (r = − 0.38, p < 0.05) was indistinct. Duration and frequency of pigs exploring the test material as well as residual material in the trough are shown in Fig. 1.

Biochar, peat and disinfectant powder were compared regarding straw as reference material. Mean exploring duration per pig was higher for biochar (63 min/day) and peat (50 min/day) than for straw (39 min/day) (p < 0.05). Mean exploring duration of disinfectant powder (24 min/day) was lower than for straw (p < 0.05) (Fig. 1-A). Mean frequency of material exploration per pig was highest for straw (39 times/day), followed by biochar (29 times/day), peat (26 times/day) and disinfectant powder (23 times/day) (p < 0.05) (Fig. 1-B). The mean amount of residual biochar (0.6 L/day) and peat (0.5 L/day) per trough was smaller than the amount of residual straw (1.0 L/day) (p < 0.05). More disinfectant powder (3.2 L/day) than straw remained in the trough (p < 0.05) (Fig. 1-C). Considering 95% and 84% confidence intervals, parameters differ significantly (p < 0.05) if 84% confidence intervals do not intersect (Fig. 1-D/E/F).

Comparing period day one and five, duration and frequency decreased (p < 0.05), whereas the residual material in the troughs increased (p < 0.05) (Fig. 1-G/H/I). However, the duration of exploring peat tended to decrease less than for the other materials (Fig. 1-G). Decrease of the frequency of material exploration was lowest for peat and lower for peat, biochar and disinfectant powder than for straw (p < 0.05) (Fig. 1-H). Residual material in the trough was lesser on day 5 than on day 1 only for peat. In contrast, more biochar, straw and disinfectant powder remained in the trough on day 5 than on day 1 (Fig. 1-I).

**Fig. 2:**
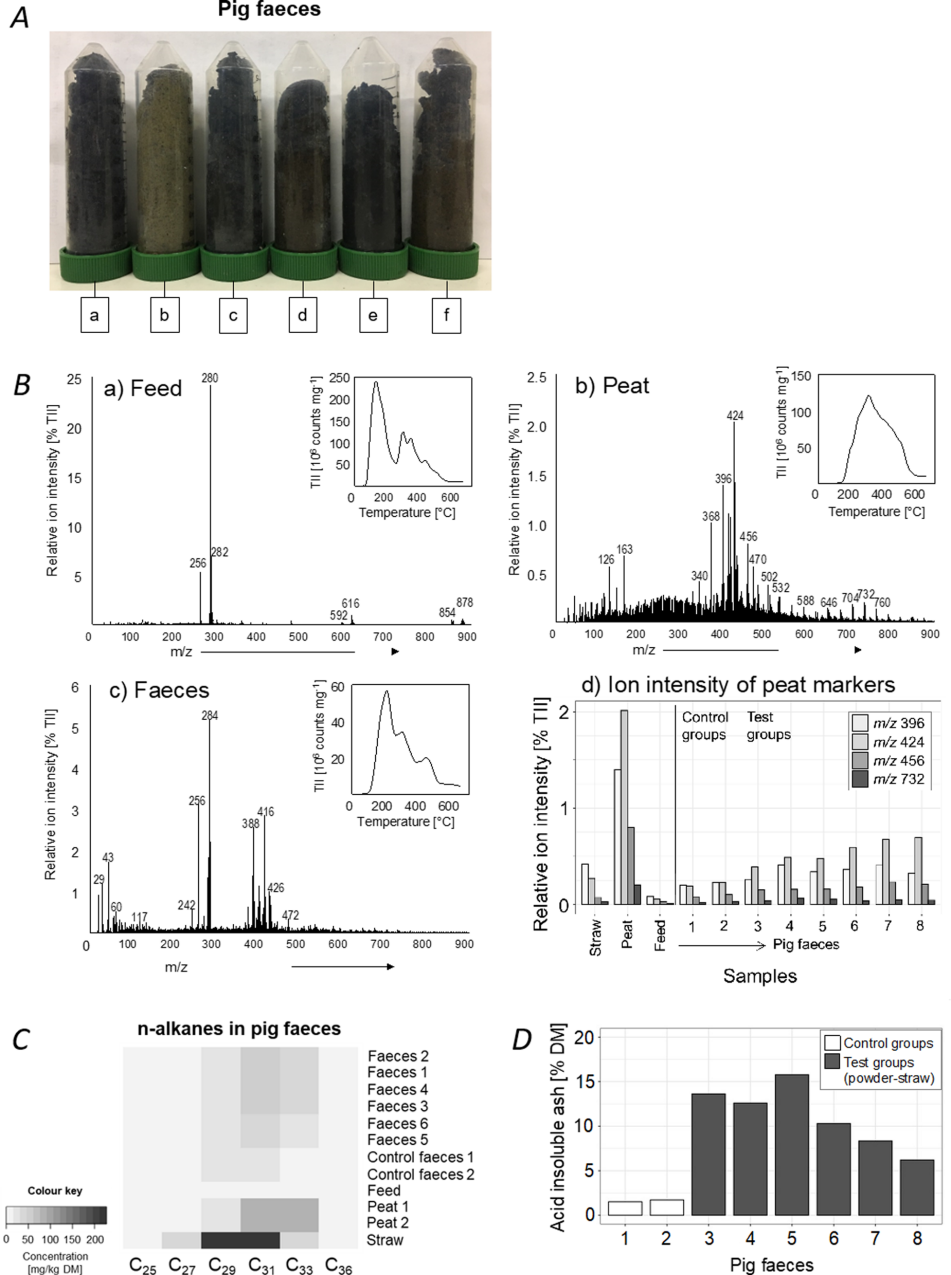
**A** Pig faeces of group one to six (period 6, day 5) receiving different material combinations: (a) powder-biochar, (b) powder-straw, (c) peat-biochar, (d) peat-straw, (e) biochar-straw, (f) powder-peat; **B** Thermograms of total ion intensity (TII) (inserts upper right) and pyrolysis-field ionization mass spectra of (a) feed, (b) peat and (c) faeces from pigs in a group receiving the material combination peat-straw; (d) shows the relative intensity of major peat marker molecular ions from Py-FI mass spectra of faces from pigs in the control groups (no material treatment) and all test groups receiving the material combination peat-straw; **C** Long-chain n-alkanes (25–36 carbon atoms) in feed, provided material (peat and straw) and faecal samples of pigs receiving no materials (Control faeces) and the material combination peat-straw (Faeces 1–6); **D** Acid insoluble ash in eight faecal samples of pigs receiving no material treatment (Control groups, sample 1 and 2) and the material treatment powder-straw (Test groups, sample 2–8)

The fact that multiple persons are involved in data collection and analysis in observational studies may result in different interpretation of behavioural elements, the duration and the frequency of exploring offered materials. To ensure quality of the results the interrater reliability was used as measure of interpretation agreement [29]. The overall interrater reliability for the two one-hour video recordings (three observers) revealed in Cohen’s Kappa of κ = 0.50, κ = 0.61 and κ = 0.61 for the two one-hour videos. For the intrarater reliability, the agreement of the two one-hour video recordings revealed in Cohen’s Kappa of κ = 0.84.

### Chemical analyses of feed, test materials and pig faeces: bulk molecular-chemical composition, n-alkanes and acid insoluble ash

According to the material combination offered to each group, the colours of the faecal samples showed clear differences (Fig. 2-A). Faeces were brown or even entirely black in groups receiving peat or biochar, respectively. Faeces of pigs receiving the powder-straw combination showed a light brown colour.

Analysing the bulk molecular-chemical composition, thermograms of total ion intensity (TII) and the Py-FI mass spectra in Fig. 2-B-a/b/c show great differences between the samples. The majority of feed sample is volatilized at a relatively low temperature of about 100–200 °C, typical for the release of fatty acids in pyrolysis field ionization mass spectroscopy (Py-FIMS). The second peak in the TII thermogram at 300 °C originates from the thermal decomposition of di- and triglycerides, and the ion intensities following at higher temperatures are from lignin building blocks. The TII thermogram of the peat sample shows the volatilization of organic matter over a wider temperature range from about 150–600 °C, peaking at 300 °C. Rather similar to feed and dissimilar to peat, the TII thermogram of the faeces sample also shows three peaks of thermal volatilization which can be assigned to fatty acids, glycerides and sterols, and lignin building blocks. The mass spectra of feed (Fig. 2-B-a) are dominated by only seven signals: *m*/*z* 256 (palmitic acid), *m*/*z* 280 (linoleic acid), *m*/*z* 282 (oleic acid), two diglycerides (*m*/*z* 592, *m*/*z* 616) and two triglycerides (*m*/*z* 854, *m*/*z* 878). Some other less intensive signals can be assigned to the thermal decomposition of peptides, and carbohydrates like cellulose and starch, which results in many signals of low intensity. The Py-FI mass spectrum of peat (Fig. 2-B-b) shows much more and different signals indicating the abundance of carbohydrates (*m*/*z* 60, 72, 84, 96, 126), phenols and lignin monomers (*m*/*z* 108, 110, 122, 124, 140), lignin dimers in the range *m*/*z* 246 to *m*/*z* 356, mostly heterocyclic N-containing compounds (*m*/*z* 59, 67, 79, 81) and peptides (e.g., *m*/*z* 57, 70, 73, 74, 75, 84, 87, 91, 97). Besides these many less abundant signals, four marker signals were particularly pronounced in the spectrum of the peat sample: *m*/*z* 396, 424, 456 and 732, which showed up neither in the feed (Fig. 2-B-a) nor in the straw sample (not shown). The abundance of these four signals in the spectrum of the faeces sample, in addition to signals of lipids (*m*/*z* 256, 284) as well as typical excrement markers like *m*/*z* 388 (coprostanol) and *m*/*z* 416 (ethylcoprostanol) form a group of intensive signals in the higher mass range (Fig. 2-B-c). Consequently, the relative ion intensities of these four peat marker signals with largest abundance in peat but much less in straw, feed and faeces from the control groups, showed a clear trend of increase in the faeces of pigs from the test compared to the control groups (Fig. 2-B-d).

N-Alkane analysis identified six homologs in a range of 25 to 36 carbon atoms in faeces of pigs, submitted peat and straw, where C_29_, C_31_ and C_33_ were most abundant. In feed, concentrations of C_25_, C_27_ and C_33_ were below the detection limit; concentrations of C_29_, C_31_ and C_36_ were between 5 and 9 mg/kg dry matter (DM). Faecal concentrations of 19–27 mg C_29_ and 22–56 mg C_31_/kg DM may resulted mainly from ingestion of straw, which had 222 mg C_29_ and 227 mg C_31_/kg DM, and in case of C_31_ from peat as well (Fig. 2-C). Peat had a low concentration of C_29_ (18 mg/kg DM) but contained distinctly more C_31_ (86–88 mg/kg DM). Peat was nearly the only material with a considerable concentration of C_33_ (77 mg/kg DM). Faecal C_33_ concentrations between 4 and 39 mg/kg DM probably showed the ingestion of peat and straw.

Analysis of AIA in pig faeces revealed a significantly higher (p < 0.05) mean content of AIA in faeces of pigs receiving the material combination powder-straw (11% DM) compared to control faeces of pigs receiving no material (1.6% DM) (Fig. 2-D). This finding is possibly due to oral uptake of disinfectant powder by pigs. Thus, the observational study and faecal analyses underpin a possible oral uptake of bedding and enrichment materials by pigs.

## Discussion

Our study revealed that pigs prefer biochar and peat over the other tested materials straw (reference material) and disinfectant powder. Analysis of pigs’ faeces for the bulk molecular-chemical composition by Py-FIMS, for n-alkanes and AIA, naturally occurring in the materials provided, confirmed a considerable consumption of peat and disinfectant powder.

Straw is the most investigated enrichment and bedding material and hence regarded as baseline material above which or below which other materials may be ranked [30]. However, previous studies identified materials others than straw, such as peat, branches, spent mushroom compost or chopped hay as more attractive to pigs [14, 15, 31]. Our investigations revealed peat, beside biochar, as the pigs’ preferred enrichment material in accordance to investigations of Beattie et al. [14] and Pedersen et al. [15]. Due to its heterogenous structure, peat stimulates the pigs’ exploratory behaviour and is further encouraged by characteristics such as being rootable, destructible, chewable and edible [30]. In contrast, Zwicker et al. [16] identified cut straw as preferred enrichment material for pigs. However, chopped straw received less attention [16].

In our results, generally time of exploring behaviour and frequency were correlated as well as duration and residual material. The mean interaction time with biochar and peat was higher than with straw (Fig. 1-A). However, the mean interaction frequency for biochar and peat was lower than for straw (Fig. 1-B). This might indicate that the motivation to manipulate peat and biochar is higher and can sustain the animals’ interest for a longer time period. Beaudoin et al. [32] investigated duration and frequency of manipulation of eight enrichment objects over a five-day period. In their study, wood had the longest mean manipulation length remaining high over the five-day period, but other objects were manipulated more frequent. Telkänranta et al. [33] investigated the manipulation frequency and detected that a high manipulation frequency with an object not necessarily involves a decreased prevalence of ear and tail biting. Consequently, frequency alone might not be representative enough regarding the attractiveness of an enrichment material to pigs [34] and the total duration of manipulation is important to estimate the enrichment quality for the animal. Hence, our data suggest that biochar and peat might be more attractive enrichment materials than straw.

Pigs quickly loose interest in enrichment materials, and exploratory behaviour is reinforced through novelty of a material or an object [35, 36]. A decrease of attention towards the materials within the five-day testing period was identified for all materials but less so for peat. Similar observations were reported for enrichment objects by Beaudoin et al. [32], who only found sustained interest of pigs in wood. This observation of decreased interest over time was confirmed in multiple studies [35–38]. Thus, enrichment material and objects need to be regularly replaced to guard their novelty effect and the interest of pigs towards the enrichment [32, 35, 36].

According to the type of material, the residual material in the trough varied. Four litres of each material per group were provided daily during the five-day testing period. Generally, less than one litre of peat remained in the trough whereas more than three litres remaining disinfectant powder were recorded in the morning prior to replenishing the trough with new material (Fig. 1-C). During manipulation, pigs either moved material to the floor outside the trough or consumed a portion thereof. Latter was especially observed for biochar and peat: on several test-days leftover material was neither documented in the trough nor on the floor. Another indication for material consumption is the colour of pigs’ faeces according to the material treatment (Fig. 2-A). No previous studies that report colour changes of pig faeces in response to the intake of several enrichment materials are known at present.

To prove material consumption, Py-FIMS is as sensitive method. Comparing the bulk molecular-chemical composition of feed, presented materials (peat, straw) and pig faeces, specific marker signals for peat could be identified in faecal samples. These spectral markers for peat at *m*/*z* 396, 424, 456 and 732 fully agree with the Py-FI mass spectra of a wider range of fen peats of different origin and management [39]. Furthermore, the mass signals of di- and triglycerides in Fig. 2-B-a have been previously detected in similar intensity ratios in Py-FI mass spectra of wheat flour (Leinweber, unpublished data). This is reflecting the composition of the diet offered to the pigs during the study, which contained wheat as a main component.

Several studies investigated the use of n-alkanes and acid insoluble ash as markers for feed intake prediction [28, 40]. Both odd- and even-chain alkanes naturally occur in the cuticular waxes of plants. In flowering plants C_29_, C_31_ and C_33_ occur predominantly [41–43], whereas leaves from deciduous trees and shrubs may also contain considerable quantities of C_27_ [44, 45]. Even-chain alkanes are usually detected with negligible concentration [46]. Cuticular wax alkanes undergo some degradation during early leaf litter degradation and during soil formation [26], which may affect their quantity and relative distribution. However, they are still present in soil or peat [47]. When ingested by an animal, alkanes might partially be absorbed in the intestine, which is why their faecal recovery is usually incomplete [40, 48]. The odd-chain alkanes detected in the enrichment and bedding materials were sensitive biomarkers to determine consumption by pigs anyhow. Especially C_33_ analysis did indicate primarily the ingestion of peat.

Titgemeyer [27] and Kavanagh et al. [28] identified AIA, which is naturally present in most feeds, as a reliable marker in digestibility studies. Kavanagh et al. [28] reported an almost complete recovery rate, whereas others found a recovery rate of about 85% [49, 50]. In our study, AIA was used as an internal marker for the oral intake of disinfectant powder. Faecal samples of pigs that received the material treatment powder-straw had a higher AIA concentration than faecal samples of those individuals that received no treatment. This provides evidence for the oral intake of disinfectant powder, as this material has a concentration of crude ash near to 100%, whereas crude ash and AIA are rather low in feed and straw (4.7 and 0.6% respectively) [51].

In our study, the bulk molecular-chemical composition, n-alkanes and AIA, naturally occurring in the provided materials, were tested as indicators for the intake of provided materials. Although no substance can fulfil all characteristics of an ideal marker [18], they verified the consumption of provided materials by pigs and can be used as a first step to identify the intake of peat and disinfectant powder by pigs.

As pigs received a balanced diet, meeting nutritional requirements (Table 1) corresponding to an ad libitum feeding regime [52], a consumption of provided materials as consequence of feed shortage can be excluded. Accordingly, Kauselmann et al. [31] found no effect of material preference on feed intake and weight gain in fattening pigs. However, the nutritional value of a material considerably contributes to its attractiveness and food feedback from a rooting material clearly increases pigs’ preference imitating a natural foraging experience [16, 31, 53, 54]. Adding for example maize kernels to straw, which has a low nutritive value and is usually less preferred than other materials [14, 15, 31], pigs’ foraging and exploration behaviour increases [16, 54]. However, feed presented exclusively, and feed mixed with rooting material were equally manipulated [53]. In the present study, possibly peat and biochar contained substances enhancing their nutritive values being attractive to pigs without necessarily complementing the diet.

**Table 1:** Nutritional composition of the experimental diet for fattening pigs

	Energy and nutrient requirements	Analysed
ME, MJ/kg	12.5–13.5^1^	14.1
DM, %		94
Crude protein, g/kg	150–200^1^	186
Crude ash, g/kg		49
Crude fibre, g/kg	4–8^2^	39
Crude fat, g/kg		26
Calcium, g/kg	5.5–7.0^1^	8.1
Phosphor, g/kg	4.0–5.5^1^	5.1
Sodium, g/kg	1.1–1.0^1^	2.1
Iron, mg/kg	50–60^2^	120
Zinc, mg/kg	50–60^3^	120
Manganese, mg/kg	20^3^	100
Copper, mg/kg	4–5^3^	55
Vitamin A, I.E./kg	2200^3^	
Vitamin D_3_, I.E./kg	150–200^3^	
Vitamin E, I.E./kg	15^3^	

^1^Jeroch et al. [61]; referred to 88% DM

^2^Flachowsky et al. [73]; referred to DM

^3^GfE [60]; referred to DM

We could show a material preference of pigs and a considerable consumption of certain materials. However, the study revealed some limitations. Material was offered in troughs and pigs might assume it is feed. Indeed, enrichment material should be edible [7]. To avoid confusion, material troughs and feeding troughs were separated. Offering material in a trough provides rootable enrichment at pig’s eyelevel and protects from soiling. In addition, material presented in a trough is readily available and accessible to the pigs’ attention. It cannot be moved to the corner of the pen or under the feeding trough [37, 55]. Hanging material, especially at pigs’ eyelevel, might attract the pigs’ attention more [37, 55], but is not rootable. Material offered on the floor, being rootable, quickly gets soiled with faeces and thus uninteresting for pigs [34, 37, 38, 56]. However, difference of interest in clean and soiled materials over short term could not be confirmed by Beaudoin et al. [32], but a possible effect over long term. Furthermore, biochar is not commonly used as enrichment material, but increasingly used with bedding material to reduce odour and gas emissions in stables and enhance fertilizing properties of animal excretions [57, 58]. However, as in our study interest of pigs in biochar is high and comparable to the interest in peat, the material might be appropriate to fulfil behavioural needs of pigs. Although no substances indicating a consumption of the respective material have been analysed for biochar, the black colour of pigs’ faeces when receiving biochar as material treatment and the absence of leftover material in the trough and on the floor on several days, respectively, could prove a material intake also for biochar.

Likewise, disinfectant powder is not commonly used as enrichment material. Its use in stables intends a hygienic aspect, principally based on the absorption of moisture, and not to fulfil behavioural needs of pigs. However, as pigs tend to explore everything in their environment, it is likely that they also investigate and possibly ingest disinfectant powders. The odour of disinfectant powders is often intense and differs from organic materials such as straw or wood and might enhance pigs’ interest [56]. Still, important characteristics (e.g. deformable, destructible), creating an attraction toward a material, are missing in disinfectant powder [56]. Consequently, the pigs’ interest in this type of material is rather low. Furthermore, due to small particle size, disinfectant powder may increase short-term dust exposure when spread in stables.

For statistical analysis, all video recordings were evaluated by the same person. Evaluating the intrarater reliability, the agreement within in the observer evaluating all videos, was 85% and revealed in Cohen’s Kappa of κ = 0.84 [29, 59]. According to Cohen [59] and McHugh’s interpretation of Cohen’s Kappa [29], this agreement is almost perfect in a range of 0.81 ≤ κ ≤ 0.99. Additionally, interrater reliability was conducted to confirm the explanatory power of the ethogram. For the three observers, this revealed in Cohen’s Kappa of κ = 0.50 to κ = 0.61 and is interpreted as moderate (0.41 ≤ κ ≤ 0.60) to substantial (0.61 ≤ κ ≤ 0.80) agreement [29, 59]. When starting the observation, the observers read the ethogram (Table 2) for the first time and were untrained in behavioural observation and untrained in using the corresponding software BORIS. Considering that, the results still indicate adequate agreement.

**Table 2:** Ethogram for observed behavioural elements

Behavioural element	Description/definition	Source (modified)
*Exploratory*		
Exploring material	Exploring the material in the trough, in the area immediately below and approximately 0.3 m (equivalent to the depth of the trough) surrounding the trough for at least 10 s, by rooting, nosing, chewing, sniffing, touching or manipulating with the snout, whilst in a standing or sitting position; if exploring the material is interrupted for 15 s or less the exploratory behaviour still counts as one behavioural element	[16, 74]
*Inactive/resting*		
Lying	Lying with eyes open or closed	[75]
Sitting	Sitting on hind quarters without exploring material, pen equipment or manipulating pen mates	[76]
*Harmful social behaviour*		
Manipulating pen mates	Nosing the belly of a pen mate or manipulating or biting the tail, ears or legs of a pen mate while standing or sitting	[16]

## Conclusions

In this study, pigs preferred peat and biochar over straw, that is generally set as gold standard for animal bedding and enrichment material. Whereas peat is generally understood as suitable enrichment material for pigs, no studies investigated the enrichment quality of biochar. Biochar in animal farming is used to improve air quality in stables and quality of manure. However, as pigs show high interest in this type of material, it might fulfil behavioural needs, and offering biochar as enrichment material might thus be a new perspective.

Chemical analysis of pig faeces revealed a considerable consumption of peat and disinfectant powder. Although no statement about the ingested quantity of materials can be made, we could show that enrichment and bedding material as well as disinfectant powders will increasingly be important in the light of feed and food safety. Future studies will address the quantification of material intake by pigs in order to estimate the risk of the transfer of undesirable substances from enrichment and bedding materials as well as disinfectant powder into the food chain.

## Materials and methods

### Experimental design

**Fig. 3:**
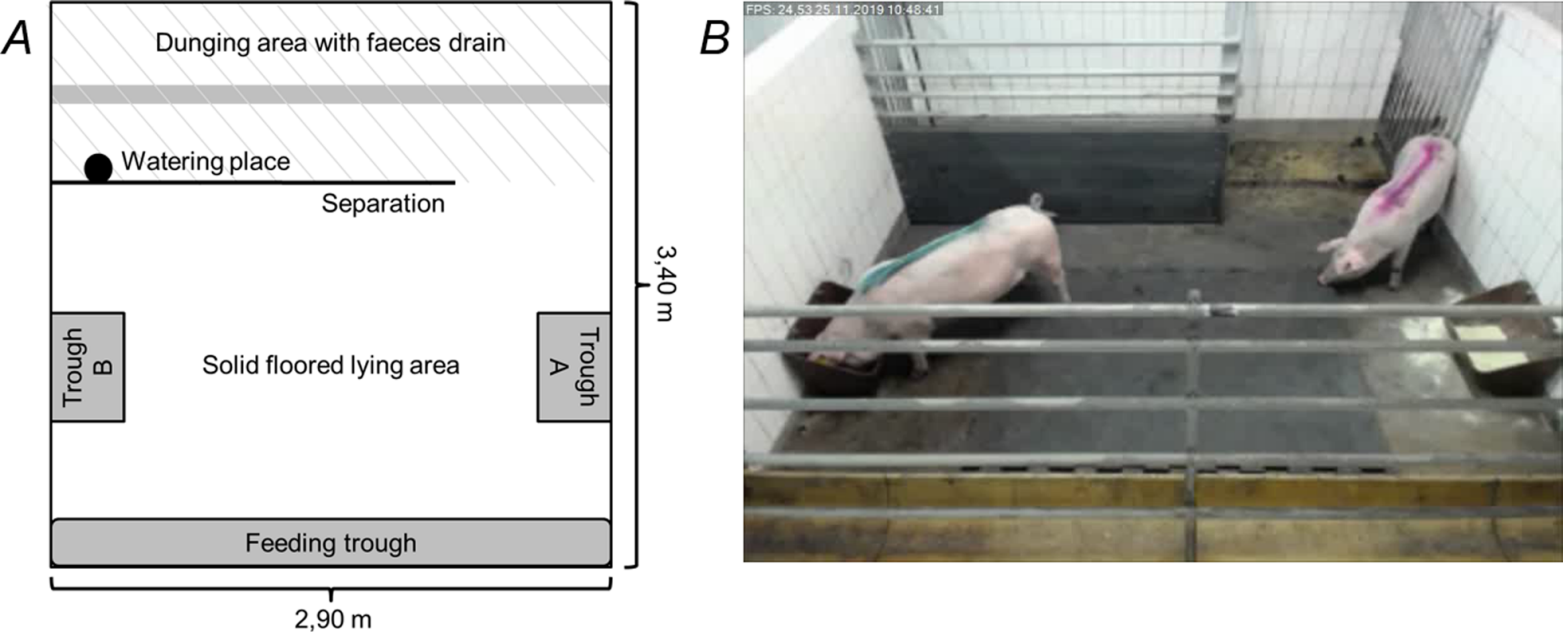
**A** Pen for a group of two pigs; two different materials were provided in trough A and B, respectively; **B** camera view from above the pen

A total of 12 female pigs (German Landrace, 26.4 ± 3.57 kg body weight) at 10 weeks of age were used in this study. Pigs were kept as pairs in six groups in identical pens (Fig. 3). They were fed a commercial diet with the daily allowance permanently adjusted to 110 g feed per kg^0.75^ bodyweight [52]. The feed (Bonimal SK Ferkel134 [BayWa AG, München, Germany]), meeting the nutritional requirements of growing pigs, was offered in three meals per day and left in the through overnight (Table 1) [60, 61].

After an adaptation period of 14 days, four materials were tested for the pigs’ preference: disinfectant powder (834 g/L), peat (308 g/L), biochar (260 g/L) and chopped wheat straw (68 g/L, defined as reference material). In a previous study, 74 materials of four categories have been analysed for their content of undesirable substances as defined for feed [12]. Accordingly, one sample of the category disinfectant powder, peat and biochar, respectively, was selected ensuring levels of toxic metals and trace elements being below maximum acceptable contents for complementary feedstuffs in case of material ingestion by pigs. The three test-materials as well as straw were purchased online.

Each pen received a different combination of two test materials (4 L of the respective material per day) over a five-day period followed by a two-day break (no material offered) before receiving a new combination. The entire preference trial lasted 42 days. The material combinations were tested in a 4 × 6 factorial arrangement considering the different test-periods in order to minimize side effects such as animal age or weather conditions (e.g. length of daylight). The materials were offered in two separate troughs located on opposite sides of the pen (Fig. 3). During the five-day observation period, two litres of fresh material were filled twice per day into each trough, respectively. Residual material was recorded in the morning prior to replenishing the troughs. To avoid habituation to one trough only, the material presentation within each pen was changed to the opposite through every morning.

### Behavioural observations

Digital cameras (Logitech C930e HD Webcam [Conrad Electronic SE, Hirschau, Germany]) were placed at each pen at approximately 2.5 m above the feeding trough. Behaviour of the pigs was recorded for seven hours per day during the five-day observation period between 9 a.m. to 1 p.m. and 2 to 5 p.m., respectively. These time frames were identified as main hours of activity of the pigs during the two-week adaptation period. Video recordings were performed in real-time mode (25 frames per second) using the software iSpy 64 v. 7.2.1.0 [62]. Explorative, inactive and harmful social behaviour (Table 2) was assessed on day one and five of each period and for each pen using the Software BORIS v. 7.8.2. (Behavioural Observation Research Interactive Software) [63].

To confirm reliability of the generated observational data, the interrater reliability as well as intrarater reliability was tested and used as measure of interpretation agreement [29]. For the interrater reliability, three scientists, untrained in behavioural observation, were asked to assess the pigs’ behaviour in two one-hour video recordings using the ethogram (Table 2). For the intrarater reliability the scientist, who assessed the pigs’ behaviour in all videos, was asked to reassess the pigs’ behaviour in two one-hour video recordings after two and seven months, respectively. Data of the three untrained scientists were compared in relation to the scientist assessing all videos. Evaluation of agreements was conducted including a ten-percentage tolerance for the time assessment and a deviation of one for frequency assessment. Tolerance levels were added as a total agreement would not be possible as time and frequency were recorded manually from each observer and frequency would count only if the pigs would explore the material at least for 10 s. As measure of agreement, Cohen’s Kappa (κ) was calculated and interpreted [29, 59].

### Chemical analyses of feed, test materials and pig faeces: bulk molecular-chemical composition, n-alkanes and acid insoluble ash

The n-alkanes and AIA naturally occur in peat and disinfectant powder, respectively. Although a low concentration of n-alkanes and AIA is also present in feed materials, the content of these substances in pig faeces may serve as a possible indicator for oral uptake of litter materials by pigs. Faecal samples of each pen were taken on day 5 of each period and the colour was photo documented. Faecal samples were stored at -20 °C for further analysis. Faecal samples collected when pigs had access to the material combination peat-straw were analysed for bulk molecular-chemical composition and n-alkanes whereas faecal samples collected when pigs had access to the material combination powder-straw were analysed for AIA. Additionally, peat, straw and feed were also analysed for bulk molecular-chemical composition and n-alkanes.

For a non-targeted **analysis of the bulk molecular-chemical composition**, Py-FIMS was conducted using 0.3 mg of freeze-dried, finely ground and homogenized samples of feed, straw, peat and faeces. The samples were thermally degraded by pyrolysis in the ion source with 4.7 kV emitter and -5.5 kV counter electrode of a double focusing Finnigan MAT 95 mass spectrometer (MasCom Technologies GmbH, Bremen, Germany). The samples were heated in a vacuum of 10^–4^ Pa from 50 to 650 °C in temperature steps of 10 K and a total pyrolysis time of 15 min. The emitter was flash heated to avoid residues of pyrolysis products between the magnetic scans. We recorded 65 spectra in the mass range between *m*/*z* 15 to 900. The Py-FIMS methodology and interpretation of marker signals (*m*/*z*) was described by Schulten and Leinweber [64].

For the **analysis of** long-chain **n-alkanes**, dry or freeze-dried samples of the test-materials, feed and faeces were ground to pass a 0.5 mm screen using a standard sample mill. Lipid extracts were obtained from the samples, purified and analysed by gas chromatography according to [65]. In brief, the samples were saponified in ethanolic potassium hydroxide for 4 h at 90 °C, extracted by phase separation into n-heptane at 75 °C, and purified through silica-gel columns. The internal standards n-docosane and n-tetratriacontane (98% purity) were priorly added to each test tube. Alkane analysis was performed on a Shimadzu GC-2010 (Shimadzu Corporation, Kyōto, Japan) fitted with a flame ionization detector and a 30 m × 0.53 mm × 0.25 µm separation column (Rtx®-1 w/Integra-Guard; Restek Corporation, Bellefonte, PA, USA). On-column injection was performed with 0.5 µL injection volume. The injection temperature programme was: 80 °C hold for 0.1 min, 100 K/min to 310 °C, then hold for 10 min. The column oven temperature programme was: 80 °C hold for 0.1 min, 50 K/min to 240 °C, hold for 1 min, 6 K/min to 264 °C, 4 K/min to 284 °C, 2 K/min to 296 °C, then hold for 10 min. Helium was used as carrier gas with 30.1 cm/s linear velocity, which was a column flow of 3.75 mL/min and a pressure of 22.7 kPa, and as makeup gas with a flow rate of 30 mL/min. An external standard solution contained a homologous sequence of the target alkanes (n-docosane to n-octatriacontane) and was used to identify the retention times and to determine the device-internal discrimination of alkanes with increasing molecular weight. Alkane concentrations were quantified on peak area basis in relation of target and internal standard alkanes and corrected for any discrimination that might have occurred during solvent extraction [66]. The n-alkanes n-pentacosane (C_25_), n-heptacosane (C_27_), n-nonacosane (C_29_), n-hentriacontane (C_31_), n-tritriacontane (C_33_) and n-hexatriacontane (C_36_) have been identified in peat, straw, feed and faecal samples.

For **analysis of AIA** in faecal samples, VDLUFA (Association of German Agricultural Analytic and Research Institutes) standard method no. 8.2 was applied [67]. In brief, 5 g of dried faecal sample material were incinerated, transferred to a beaker and boiled with 75 ml 3 N-hydrochlorid acid for 15 min. The solution was filtered through ashless filter paper with hot water until free of acid. Finally, filter and residue were dried and incinerated at a temperature of 550 to 650 °C.

### Analyses of the nutritional composition of the diet

Feed was analysed for DM, crude protein, crude ash, crude fibre and crude fat (Weender Analysis) as well as starch (polarimetric method) following Commission Regulation (EC) No 152/2009 [68]. Analyses of bulk elements calcium, phosphor and sodium as well as trace elements iron, zinc, manganese and copper were performed after microwave-assisted acid digestion with nitric acid (65%) using inductively coupled plasma mass spectrometry according to DIN EN ISO 17294-2:2017-01.

### Statistical analyses

To define the relation between variables, the Pearson correlation coefficients were computed between exploring duration, exploring frequency and residual material, respectively. In order to take important factors into account, linear mixed models were fitted using R 4.0.4 with packages lme4 and lmerTest [69–71]. Duration and frequency of observed exploring behaviour (Table 2) were summarized as an average value per pig and day. Residual material in the trough was summarized as a mean value per pen and day. Focus material (material that is currently explored by the pig), period day (with interactions) and secondary material (material that is not currently explored by the pig) were treated as fixed effects. Period and pig were treated as random effects. Chopped straw was regarded as reference material. From the model coefficients and standard error, 95 and 84% confidence intervals were computed. The 95% confidence intervals are useful because a parameter differs significantly (at the 5% level) from zero if and only if its 95% confidence intervals does not contain the value 0. The 84% confidence intervals are useful because two parameters differ significantly (at the 5% level) if and only if their 84% confidence intervals do not intersect [72]. Therefore, the representation of confidence intervals opens a way of visually presenting the differences of parameter estimates without over-stressing the results of multiple testing. All statistical conclusions were based on these model coefficients with confidence intervals and the respective p-values.

## Data Availability

The datasets generated and analysed during the current study are available from the corresponding author on reasonable request.

## Abbreviations

AIA: Acid insoluble ash
DM: Dry matter
EU: European Union
Py-FIMS: Pyrolysis field ionization mass spectroscopy
TII: Total ion intensity
VDLUFA: Association of German Agricultural Analytic and Research Institutes
